# An analysis of cigarette sales during Poland’s menthol cigarette sales ban: small effects with large policy implications

**DOI:** 10.1093/eurpub/ckac063

**Published:** 2022-06-09

**Authors:** Alex C Liber, Michal Stoklosa, David T Levy, Luz María Sánchez-Romero, Christopher J Cadham, Michael F Pesko

**Affiliations:** Georgetown University-Lombardi Comprehensive Cancer Center, Cancer Prevention and Control Program, Washington, DC, USA; Institute for Health Research and Policy, University of Illinois at Chicago, Chicago, IL, USA; Department of Public Health and Social Medicine, Medical University of Gdańsk, Gdańsk, Poland; Georgetown University-Lombardi Comprehensive Cancer Center, Cancer Prevention and Control Program, Washington, DC, USA; Georgetown University-Lombardi Comprehensive Cancer Center, Cancer Prevention and Control Program, Washington, DC, USA; Department of Health Management and Policy, University of Michigan School of Public Health, Ann Arbor, MI, USA; Department of Economics, Georgia State University Andrew Young School of Public Policy Studies, Atlanta, GA, USA

## Abstract

**Background:**

In May 2020, the European Union Tobacco Products Directive mandated that EU member states, including Poland, ban the sale of menthol cigarettes. With menthol making up 28% of cigarette sales before the ban, Poland is the country with likely the largest menthol cigarette sales share in the world to ban their sale. We analyze how this ban changed the Polish tobacco market.

**Methods:**

We use monthly NielsenIQ data (May 2018–April 2021) on sales of cigarettes and roll-your-own tobacco by menthol and standard flavor in eight regions of Poland. We set up a bite-style regression model controlling for pre-ban menthol share, climate, border opening status, and Apple movement data to estimate the effect of the May 2020 menthol ban.

**Results:**

We find menthol cigarette sales fell at least 97% after the menthol cigarette ban across Poland and standard cigarette sales replaced them. Regression modeling indicates that total cigarette sales fell, after the ban, an average of 2.2 sticks per capita per month, equal to a 2.9% decline, however, results were not significant (*P* = 0.199). The bite component of our model reveals total cigarette sales did decline significantly in the regions with the highest pre-ban menthol sales shares. Roll-your-own tobacco sales increased by a statistically insignificant 0.03 stick-equivalents after the ban (*P* = 0.798). Product prices also fell in the wake of the menthol ban.

**Conclusions:**

In Poland, the EU state with the one of the largest pre-ban menthol shares, we find mixed evidence that the ban is working as intended.

## Introduction

To fulfill the European Union’s (EU) 2012 Revised Tobacco Products Directive (TPD), on May 20, 2020, Poland and 27 other EU member countries banned the sale of menthol cigarettes.^1^ This policy intended to decrease initiation into cigarette smoking by banning a chemical masking the harshness of cigarette smoke and thereby averting some portion of the massive morbidity and mortality exacted by tobacco use in the EU.^2^ All other characterizing flavors except menthol had been previously removed from cigarettes and roll-your-own (RYO) tobacco sold in May 2016.^3^ Before the 2020 menthol ban, menthol cigarettes were sold in two primary forms; cigarettes whose tobacco leaf is coated in menthol and cigarettes containing a crushable mentholated liquid capsule in the filter.^4^ Both were banned, in addition to menthol RYO tobacco, in 2020. Before implementation, Poland challenged the menthol cigarette ban at the European Court of Justice, arguing it created unreasonable trade barriers.^5^ That court ruled against Poland, maintaining the TPD was designed to protect public health and ruling that banning the sale of menthol cigarettes could proceed.^6^

Public health experts expect menthol cigarette users to exhibit four behaviors in reaction to a menthol cigarette ban: (i) switch to non-mentholated cigarettes (which could be self-mentholated with flavoring cards or menthol capsules), (ii) quit smoking cigarettes altogether, (iii) switch to using mentholated or non-mentholated non-cigarette products (electronic cigarettes and heated tobacco products), and (iv) continue using menthol cigarettes obtained through illicit sources.^2^ These hypotheses are generally based on emerging evidence from countries such as Canada, which banned menthol cigarettes nationwide in 2017, or the results of stated preference surveys of menthol users.^7–9^ Still, findings derived largely from Canada may not generalize to other countries with much larger menthol cigarette shares that exceed one-quarter of sales like Poland, USA, Singapore, and the Philippines.^10^ Results such as these provide some insights into the potential effects of a menthol ban. Still, the relative size of the group pursuing each of the four post-ban behaviors among pre-ban smokers is not yet established in the scientific literature.

Thus far, little research has evaluated the effects of the EU TPD’s menthol cigarette ban on the tobacco market of member countries beyond a survey of menthol cigarette smokers in England and a census of tobacco company communication to retailers in the Czech Republic.^11^^,^^12^ Prior research has found that the 2016 ban on non-menthol characterizing flavors in cigarettes significantly decreased smoking among those who had previously smoked flavored products.^13^ According to Euromonitor data (with the caveat that these data are created in collaboration with the tobacco industry),^14^ sales of menthol cigarettes in the EU as a whole were comparable to Canada—7% of all cigarettes were mentholated before the EU TPD policy—in Poland, menthol cigarette sales shares were 28%, the highest of any EU country.^10^ Before the 2020 menthol ban, some 30% of adults in Poland smoked cigarettes, and around 22% of cigarette users claimed to primarily smoke menthol cigarettes.^15^ These menthol cigarette smokers tended to be female, younger, better educated, and wealthier than other smokers.^16^ Other data points to the possibility that similar levels of menthol cigarette consumption took place in other EU countries.^15^^,^^16^ There is good reason to think that Poland is one of the first countries with a large menthol cigarette market to attempt to ban sales of those products.

Without understanding the relative distribution of users’ behaviors in reaction to a real-world ban on the sale of menthol cigarettes, we cannot accurately estimate the public health impact of such menthol bans. In this paper, we describe changes in tobacco sales in Poland after the menthol ban came into effect in May 2020. We exploit differences in the pre-ban share of menthol cigarettes to determine if the menthol ban decreased total cigarette sales.

## Methods

We utilize data on the sales of cigarettes and RYO tobacco collected by NielsenIQ from May 2018 to April 2021. Sales were separated by flavor (standard and menthol) for each product (cigarettes and RYO) and across eight Nielsen-designated regions in the country (Warsaw City, Central, West, East, North, South, South-East, South-West). Sales data were collected from grocery stores, discounters, hypermarkets, liquor stores, kiosks, petrol stations, and tobacconists. In 2019, Nielsen data covered 80% of Poland’s cigarette sales and 70% of RYO tobacco sales.^17^

As shown in table 1, the Nielsen regions align with the combined borders of first-level administrative divisions of Poland, except that Warsaw is separated from its larger region. Sales are adjusted for 2018 population levels and reported on a sticks-per-capita, per-month basis. One cigarette is equivalent to one stick, and 0.75 g of RYO tobacco is one stick-equivalent.^18^ Prices are adjusted for inflation to May 2018 levels.^19^

**Table 1:** Nielsen region details

Nielsen region	Poland’s first-level administrative divisions	May 2018 menthol share	International borders
*Central*	Mazowieckie, Łódzkie	27.84%	None
*East*	Warmińsko-Mazurskie, Podlaskie, Lubelskie	27.99%	Russia, **Lithuania**, Belarus, Ukraine
*North*	Zachodniopomorskie, Pomorskie, Kujawsko-Pomorskie	25.80%	**Germany**
*South*	Śląskie	26.78%	**Czechia**, **Slovakia**
*South East*	Świętokrzyskie, Małopolskie, Podkarpackie	28.25%	**Slovakia**, Ukraine
*South West*	Dolnośląskie, Opolskie	26.08%	**Germany**, **Czechia**
*Warsaw*	Warsaw City	36.84%	None
*West*	Lubuskie, Wielkopolskie	25.43%	**Germany**

Notes: **Bolded** countries are EU Members*.* Warsaw is not a first-level administrative division. It is located within the first-level administrative division of Mazowieckie. Nielsen, however, separately reports sales in Warsaw separately from its larger region.

Since the EU TPD policy was implemented in Poland at a national level, it is difficult to know if any effects of the policy on tobacco purchases were driven by the ban or other shocks occurring nationally at the same time. As an alternative, we compare the ban’s effects on cigarette purchases in Poland’s regions with different menthol cigarette sales shares before the ban (see shares in table 1). We expect to find greater effects of the menthol ban in Poland regions with more pre-TPD mentholated cigarette use. By leveraging differences in the baseline (May 2018) menthol sales rate by region (ranging from 25.4% of sales by value in the Western region to 36.8% in Warsaw) and interacting this with the menthol ban, we examine whether those regions with more menthol cigarette sales before the ban observe larger changes in sales after the menthol ban. This reduces concerns that co-occurring policies could confound our estimates unless these policies are also correlated with the baseline menthol share. This ‘bite’-style model is a form of a dose-response difference-in-differences model used to study other health policy changes.^20^^,^^21^

An indicator variable captures the menthol ban effect for the proportion of each month that the ban was in place. Since the first month is used to form the menthol share ‘bite’ variable, this month is excluded from the multivariate analyses. The ‘bite’ variable stands in to measure the amount of treatment or intervention applied to each region. If the bite value is larger, we should see more policy effect.

We estimate a generalized linear model using maximum likelihood estimators with standard errors clustered at the region level at which our bite variable, the menthol share at baseline, varies. The model also includes regional fixed-effects to control for time-invariant variables within regions and a continuous time trend to control for variables that vary over time linearly but not across regions. We applied the following equation:

(1) $$\;{\mathrm{Per}\;\mathrm{Capita}\;\mathrm{Cigarette}\;\mathrm{Sales}}_{it}=\;\alpha +\beta_{1}\mathrm{Menthol}\;\mathrm{Ban}+\beta_{2}\left({\mathrm{Menthol}\;\mathrm{Ban}}_{t}\times {\mathrm{May}\;2018\;\mathrm{Menthol}\;\mathrm{Share}}_{i}\right)+\beta_{3}{\mathrm{Heating}\;\mathrm{Degree}\;\mathrm{Days}}_{it}+\beta_{4}{\mathrm{EU}\;\mathrm{Border}\;\mathrm{Status}}_{it}+\beta_{5}{\mathrm{Non}\;\mathrm{EU}\;\mathrm{Border}\;\mathrm{Status}}_{it}+\beta_{6}{\mathrm{Walking}}_{it}+\beta_{7}{\mathrm{Employment}\;\mathrm{Rate}}_{it}+\beta_{8}{\mathrm{Price}}_{it}+\omega_{t}+\theta_{i}+\epsilon_{it}$$

where *i* indexes regions of Poland and *t* indexes the year-by-month from June 2018 to April 2021. For ease of interpretation, the May 2018 Menthol Share is indexed to 1 for the average share of 28.1%. The primary outcome of interest, the effect of the menthol ban in a region with an average-sized menthol share in the baseline period, is obtained by summing β_1_ and β_2_.

We attempt to control for additional sources of variation with additional covariate controls. For the economic environment, we use the quarterly employment rate in each Nielsen region.^22^ For changing weather patterns, which have been found to affect cigarette sales,^23^ we use the average monthly proportion of heating degree days (days below 10°C) in each Nielsen region.^24^

We also control for several other co-occurring changes related to the first wave of the COVID pandemic hitting the country, which coincided with the introduction of the EU TPD menthol cigarette ban. To control for the closure of international borders,^25^ we calculate an EU border open and a non-EU border open variable for each region (see values in Supplementary figure S1). The variables are equal to the proportion of each month in each Nielsen region that Poland and each EU member state’s or non-EU member states’ borders were open to bi-directional travel. For the South and South-West regions which border two EU countries, this proportion was averaged each month between both borders. For regions without an external border, this value was set to zero. We expect closing borders with non-EU countries to cause sales of cigarettes in Poland to rise as illicit cigarette channels from Ukraine, Belarus, and Russia are cut off.^26^ We expect that closing EU borders will be associated with a fall in cigarette purchases. In 2020, Poland had the least expensive cigarettes among its EU neighbors, and price-motivated cross-border purchasing activity into Poland should decline.^17^ Further, we attempt to control changes in mobility during the COVID pandemic using average monthly data on walking and driving movements at the regional level from Apple Maps.^27^ These data have been used to study the spread of COIVD around the globe.^28^

We also consider prices for cigarettes and RYO by dividing the inflation-adjusted monetary value of sales by the volume of sales for each region and flavor of product. We control for this in some specifications of the model, in which we implicitly assume that prices are not impacted in any way by the menthol flavor ban. However, the menthol flavor ban may impact the average prices paid for all cigarettes because mentholated cigarette prices were more expensive than non-mentholated cigarette prices (see Supplementary figure S2). Additionally, because the menthol ban may have affected the demand for cigarettes that we observe in the Nielsen data, prices may have responded accordingly. Alternatively, if the industry is operating in a less-than-fully competitive environment, they may have sufficient market power to set prices and respond to the menthol ban by lowering prices to prevent losing customers.

Before we proceed with analyzing the full model, we test whether it is suitable to determine if localities with more menthol share before the menthol ban had similar trends before the policy change than localities with less menthol share (e.g. the ‘parallel trends’ assumption). We conducted an event study by replacing the event of the menthol ban in the previous equation with a year-by-month time indicator to study how cigarette sales changed over time in localities with higher menthol share compared with lower menthol share. Ideally, coefficients in the pre-ban period are relatively small, suggesting no differential trending across regions before the ban.

We add an analysis of RYO sales (using per-capita stick equivalents) to assess whether smokers may have substituted towards those products after the menthol ban. Mentholated filters, crush ball filters, and mentholated rolling papers were introduced by tobacco companies in the months leading up to the menthol ban and could be a source of legally mentholated cigarettes after the ban.^29^ We evaluate model fit by minimizing Akaike information criterion (AIC) and Bayesian information criterion (BIC) values. We also report model results without price, as prices declined soon after the menthol ban, and observing the effect of that variable’s inclusion is important to evaluate the findings.

## Results

### Descriptive statistics

**Figure 1:**
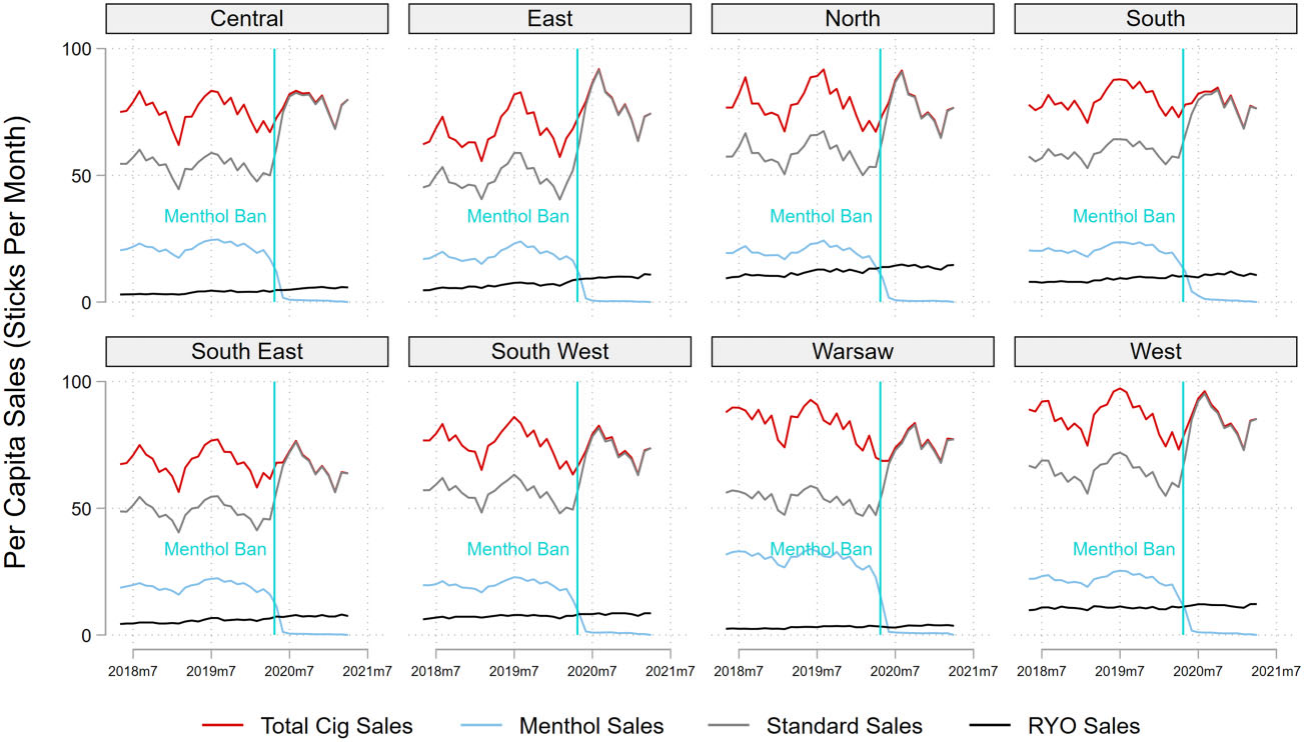
Per capita cigarette sales by Nielsen region (May 2018–April 2021). Notes: 1st quartile (lowest) menthol share regions=North and West; 4th quartile (highest) menthol share regions=South East and Warsaw. Standard, unflavored cigarettes

Figure 1 illustrates the per capita cigarette and RYO sales pattern by region across Poland from May 2018 to April 2021. Sales of menthol cigarettes declined by 97% after the ban, while sales of unflavored ‘standard’ cigarettes rose on average by 38% in their place. Total cigarette sales appear to follow the same seasonal sales patterns after the ban as they did before, making a level change in sales difficult to detect. RYO sales appear to grow over time, but the rate of change seems to vary across regions. Supplementary figure S2 shows that prices for menthol cigarettes remained higher than standard cigarettes and RYO tobacco throughout the study period in every region. After the menthol ban, as menthol cigarette sales dwindled, the total cigarette price converged with standard cigarette prices. It then begins a steady decline through the end of the study period. Table 2 displays the unweighted mean of model covariates before and after the implementation of the menthol ban split between regions by 1st quartile (Low), interquartile range (IQR), and 4th quartile (High) by May 2018 menthol share.

**Table 2:** Average monthly model covariate values before and after menthol ban implementation in regions with 1st quartile, interquartile range (IQR) and 4th quartile May 2018 menthol shares

	Before menthol ban					After menthol ban				
	Mean			*P*-value		Mean			*P*-value	
	1st Qtile	IQR	4th Qtile	1st vs IQR	IQR vs 4th	1st Qtile	IQR	4th Qtile	1st vs IQR	IQR vs 4th
Per capita menthol cigarette sticks sold	20.934	20.338	24.851	0.404	<0.001	1.413	1.639	1.518	0.779	0.881
Per capita standard cigarette sticks sold	61.358	53.868	51.154	<0.001	0.007	80.084	76.615	69.466	0.080	<0.001
Per capita total cigarette sticks sold	82.292	74.206	76.005	<0.001	0.253	81.496	78.254	70.984	0.060	<0.001
Per capita total RYO sticks sold	11.039	6.315	4.197	<0.001	<0.001	12.865	8.655	5.529	<0.001	<0.001
Menthol cigarette stick price (Real PLN)	0.713	0.709	0.722	0.016	<0.001	0.742	0.727	0.755	0.018	<0.001
Standard cigarette stick price (Real PLN)	0.688	0.683	0.696	0.010	<0.001	0.696	0.689	0.702	0.035	<0.001
Total cigarette stick price (Real PLN)	0.694	0.690	0.704	0.020	<0.001	0.697	0.690	0.702	0.039	<0.001
Total RYO stick price (Real PLN)	0.461	0.462	0.475	0.852	0.018	0.472	0.467	0.475	0.579	0.335
Employment rate (%)	56.802	55.279	57.558	<0.001	<0.001	56.335	54.992	57.035	0.025	0.001
Heating degree days (%)	0.240	0.279	0.262	0.426	0.724	0.296	0.336	0.320	0.650	0.858
EU border open (%)	0.935	0.623	0.466	<0.001	0.052	0.878	0.585	0.439	0.014	0.215
Non-EU border open (%)	0.000	0.305	0.468	<0.001	0.030	0.000	0.017	0.078	0.660	0.126
Walking volume	97.130	95.681	96.516	0.632	0.782	128.49	104.42	69.724	0.028	0.002

Notes: May 2020 is included in the After-ban group. 1st quartile (lowest) menthol share regions=North and West; 4th quartile (highest) menthol share regions=South East and Warsaw; Remaining are in IQR. Menthol share groupings throughout this table correspond to sales in May 2018.

Table 2 also reports comparison-of-means test *P*-values corresponding to whether the 1st and 4th menthol share quartile regions had average values significantly different from the IQR regions before and after the menthol ban. Table 2 shows that before the menthol ban, in regions with the highest shares of menthol sales, per capita sales of menthol cigarettes were significantly higher (*P* < 0.001) than in counterparts with fewer menthol sales. Standard cigarette sales were significantly lower in those regions with the most menthol share before the ban (*P* = 0.007). Total cigarette sales were highest in regions with the lowest menthol shares before the ban (*P* < 0.001), but similar when comparing the highest menthol share regions to those in the IQR (*P* = 0.253).

After the ban, there was no significant difference in menthol cigarette sales between the quartiles (both, *P* > 0.779). Standard cigarette sales rose across the board. Unadjusted results show that total cigarette sales fall by five sticks per capita per month in the highest (4th quartile) pre-ban menthol share regions while rising by four sticks per capita in the IQR and falling by less than one stick per capita in the lowest (1st) quartile. While the data presented in table 2 suggest that regions with the highest menthol sales before the ban had the largest reduction in total cigarette sales after the menthol ban, several other control variables changed meaningfully before and after the menthol flavor ban. Therefore, a fully adjusted regression model using a more precise measure of the pre-ban menthol cigarette share is important to study this relationship more accurately.

### Multivariate results

Regression models examine the effect of the menthol ban on cigarette sales in table 3. For ease of interpretation, at the bottom of table 3, we display the menthol ban’s average effect size and *P*-values for a region with an average pre-ban menthol share. We see that although the menthol ban seems to be associated with a small decrease in total cigarette sales (Effect without Price control −2.15 sticks per person per month [95% CI −5.43 to 1.13], Effect with Price control −1.03 sticks per person per month [95% CI −4.51 to 2.44]), this effect is not statistically significant at the 95% confidence level. However, we find that the larger the pre-ban menthol share was in a region, the larger the decline in total cigarette sales after the ban. In Supplementary figure S3, we display the predicted sales split out by pre-ban menthol share and note that in the region with the highest pre-ban menthol share (Warsaw), we observed a statistically significant decline in sales after the menthol ban. We also detect a non-statistically significant increase in the sales of RYO tobacco for an average pre-ban menthol share region (Effect without Price control −0.03 stick equivalents per person per month [95% CI −0.18 to 0.24], Effect with Price control −0.03 stick equivalents per person per month [95% CI −0.29 to 0.35]).

**Table 3:** Fully adjusted regression results table for per month per capita total cigarettes and roll-your-own tobacco

	(1)	(2)	(3)	(4)
	Cigarettes	Cig + Price	RYO	RYO + Price
Post	12.87^*^ [2.893,22.85]	15.83^**^ [4.862,26.80]	2.312^*^ [0.334,4.291]	2.320^*^ [0.441,4.199]
Pre-menthol share # post	−15.02^***^ [−22.49, −7.558]	−16.86^***^ [−24.94, −8.784]	−2.285^*^ [−4.304, −0.266]	−2.288^*^ [−4.266, −0.309]
Time trend	0.0908 [−0.0696,0.251]	0.0314 [−0.142,0.205]	0.0883^***^ [0.0558,0.121]	0.0881^***^ [0.0517,0.125]
Heating degree days	−13.15^***^ [−15.56, −10.74]	−14.26^***^ [−16.26, −12.25]	−0.574^***^ [−0.811, −0.337]	−0.575^***^ [−0.812, −0.337]
EU border open	8.446^***^ [6.226,10.67]	4.528^**^ [1.606,7.450]	0.0103 [−0.729,0.749]	0.00536 [−0.736,0.747]
Non-EU border open	−6.766^*^ [−13.52, −0.00966]	−7.371^*^ [−13.17, −1.567]	−1.110^*^ [−2.119, −0.101]	−1.110^*^ [−2.119, −0.101]
Walking volume	3.695^***^ [1.678,5.711]	4.939^***^ [2.962,6.915]	0.222 [−0.238,0.682]	0.222 [−0.243,0.687]
Employment rate	0.865^*^ [0.00475,1.726]	1.380^***^ [0.667,2.092]	0.105 [−0.0826,0.294]	0.107 [−0.0901,0.304]
Average cigarette price per pack (PLN real)		−7.594^***^ [−10.57, −4.623]		
Average RYO price per stick Eq. (PLN real)				−0.118 [−4.957,4.722]
Constant	−15.29 [−151.4,120.8]	154.1 [−30.49,338.8]	13.38 [−8.435,35.19]	13.64 [−13.26,40.54]
Observations	272	272	272	272
Mean menthol ban effect size	−2.150	−1.032	0.0272	0.0326
Mean menthol ban effect *P*-value	0.199	0.561	0.798	0.841

Notes: Region-fixed-effects coefficients are suppressed for space considerations. Mean menthol ban effect size is equal to the sum of the post coefficient and the interaction of average pre-ban menthol share and the post coefficient. RYO, roll-your-own. 95% confidence intervals in brackets;

* *P *<* *0.05,

** *P *<* *0.01,

*** *P *<* *0.001.

Supplementary figures S3 and S4 show that cigarettes and RYO tobacco sales did not trend differently before the ban in regions with higher menthol share (i.e. meeting the ‘parallel trends assumption’). In the post-period, there was a sharp temporary reduction in cigarette sales that appears to have dissipated after three months. The table 3 analysis confirms this decline, as the interaction between menthol shares and the post-ban variable is significant and negative for cigarettes and RYO. This indicates the post-ban decline in cigarettes, and RYO sales were steeper in regions with higher pre-ban shares of menthol cigarette sales. We can also rule out the possibility that more flavored RYO products were purchased as only 0.4% of RYO tobacco in Poland was flavored before the menthol ban, while that figure declined to 0.3% of all RYO sales after the ban (even though these products were still subject to the same 2016 ban on characterizing flavors as cigarettes).

Our models identified various other statistically significant covariates, as shown in table 3. RYO sales increased significantly over time, while cigarette sales were unchanged. Colder months, where heating degree days were more common, had significantly lower cigarette and RYO sales. The employment rate and the Apple Walking index were consistently associated with significantly higher cigarette sales. Having an open EU border was associated with significantly higher cigarette sales while having an open non-EU border was consistently associated with significantly lower cigarette sales. Higher prices were associated with lower cigarette sales, while the effect of RYO price on RYO sales was not statistically significant.

## Discussion

We found no significant change in the sale of cigarettes in Poland attributable to the menthol ban. Our bite-style analytical design lets us parse whether those regions with more prior exposure to menthol sales saw larger changes after the menthol ban. We find that regions with more menthol share before the ban, like Warsaw, saw a significant reduction in total cigarette sales. Regions with sub-average baseline menthol cigarette share did not see significant declines. These limited effects resulted in a non-statistically significant reduction in cigarette sales overall.

Cross-border sales effects proved to be significant in the analysis. Our findings indicate that COVID-related border closures altered regional sales flows in licit and illicit markets. Changes in movement during COVID also provided a source of regional variation that was significantly associated with cigarette sales.

This study adds evidence to a growing list of research about the effects of banning the sale of flavored tobacco products in jurisdictions where the sale of such products is quite substantial. Notably, evidence has emerged from San Francisco, USA, detailing the effect of that city’s efforts to ban the sale of flavored tobacco products that made up nearly 40% of sales before the policy change. Studies using sales data suggest decreases in all flavored tobacco products occurred, without significant substitution towards jurisdictions still selling flavored products.^30^ However, a population tobacco use prevalence study found that youth cigarette smoking increased after the San Francisco ban effective date.^31^ Our study adds to evidence that the large-scale dislocation of consumers seeking to replace menthol cigarette sales in Poland and elsewhere requires close study and careful implementation to ensure that such flavored tobacco sales bans produce positive public health outcomes.

The Poland example also provides insights into the responses by the industry to these new restrictions. Industry tactics to evade the menthol ban across Europe were widely reported to be taking place in the popular press.^29^ Imperial Tobacco released a line of cardboard cigarette pack inserts to impart a menthol flavor to standard cigarettes. As seen in other jurisdictions, Japan Tobacco International released menthol cigarillos under the same brand name as formerly mentholated cigarettes and renamed formerly mentholated cigarettes as ‘green’ editions implying through color that they still tasted like their mentholated forebearers.^32^ Philip Morris International and British American Tobacco courted retailers fearing losing sales to stock their still-legal flavored, heated tobacco and e-cigarette products.^12^ Our finding that the menthol cigarette ban had limited success may be due to these industry actions.

By selling and using products that impart a menthol flavor, by implying the presence of a flavoring capsule or cooling flavors through package graphics, or by selling through the introduction of TPD-exempt cigarillos with characterizing flavors, commercial entities and consumers alike have attempted to evade the intended effects of the menthol ban.^8^^,^^33^^,^^34^ These strategic workarounds could raise costs and inconvenience for consumers of smoking mentholated cigarettes, but since non-mentholated cigarettes were cheaper than mentholated cigarettes in Poland before the ban it is not clear if these workarounds result in higher or lower overall costs to smoke. Nonetheless, these evasion tactics should give pause to those who expect to see certain public health outcomes after a menthol cigarette ban. Reflecting on the best policy responses to the techniques is necessary for EU countries and other jurisdictions considering banning menthol cigarette sales.

### Limitations

Our findings are not without limitation. We cannot examine whether there was a differential substitution to flavored tobacco products that were not covered by the EU TPD menthol ban. Nielsen could not provide region-level data on sales of flavored e-cigarettes, cigars, or heated tobacco products that continued to be sold after the EU menthol ban. Survey research from USA finds that cigarette smokers claim they are interested in switching to these products after a flavor ban,^2^ but we cannot observe it here. Further work must determine if substitution happened to these products as a real-life menthol ban played out across Europe.

We were also unable to track whether consumers moved to illicit cigarette sources after the ban. However, we attempted to control for access to illicit cigarette sources by using border variables. Also, we have an incomplete understanding of movement trends before COVID. The Apple mobility data were only provided after February 2020, so we cannot fully understand the role of movement in the pre-COVID period. Future efforts to understand the role of movement and cigarette sales would allow us to better disentangle how this intriguing variable operates going forward.

Additionally, we cannot track the sales, prices, and usage patterns of menthol-imparting or menthol-ban evading products to determine if they are truly undermining the implementation of a menthol ban. This should be the subject of future research.

## Conclusions

After a protracted 8-year delay between the passage of the EU TPD and the implementation of the menthol ban, a great deal of change came to the Polish cigarette market in 2020. In the EU country that was likely most exposed to the bloc’s menthol cigarette sales ban, total cigarette sales have not significantly changed. Most of the initial reduction in cigarette sales because of the menthol ban was lost within three months. Other jurisdictions considering menthol cigarette bans may wish to proceed cautiously to maximize policy effectiveness. Questions relating to policy implementation and enforcement remain regarding translating an initial reduction in cigarette sales into permanent reductions.

## Supplementary data

Supplementary data are available at *EURPUB* online.

## Funding

This study was supported by the Norwegian Cancer Society in the framework of a project partnership with the Polish Ministry of Health by leveraging funding from Norway Grants (Donor Partnership Project No. NMF.PL-NOR.DOI.PDP2_2/20//3725/2020/80) and by the US National Cancer Institute (NCI) and the US Food and Drug Administration (FDA) through grant U54CA229974.

*Conflicts of interest*: None declared.

Key points

- Studies of the effects of menthol cigarette bans have been confined to jurisdictions where menthol cigarette sales were small before a ban or to artificial laboratory settings.
- Using a bite-style difference-in-differences model, we study the effect of Poland’s menthol ban, the country with the highest level of menthol cigarette sales share to yet attempt a ban on sales of menthol cigarettes.
- We find total cigarette sales did not significantly decline after the menthol cigarette ban.
- The evidence from Poland indicates that a menthol cigarette ban on its own may not cause a sizeable decline in cigarette sales, further pointing to the need for other jurisdictions considering banning menthol from cigarettes to study those conditions that might have blunted the impact of what was anticipated to be a significant public health policy advance.

## Supplementary Material

ckac063_Supplementary_Data
