# Supplementary material for: An analysis of cigarette sales during Poland’s menthol cigarette sales ban: small effects with large policy implications
Source: Eur J Public Health. 2022 Jun 9;32(5):735–40. doi: 10.1093/eurpub/ckac063 (PMC9527965; doi:10.1093/eurpub/ckac063)
Supplement: ckac063_Supplementary_Data [file ckac063_supplementary_data.docx]

# Supplemental Material

Supplemental Figure 1: Regional Border Opening Status (May 2018—April 2021)


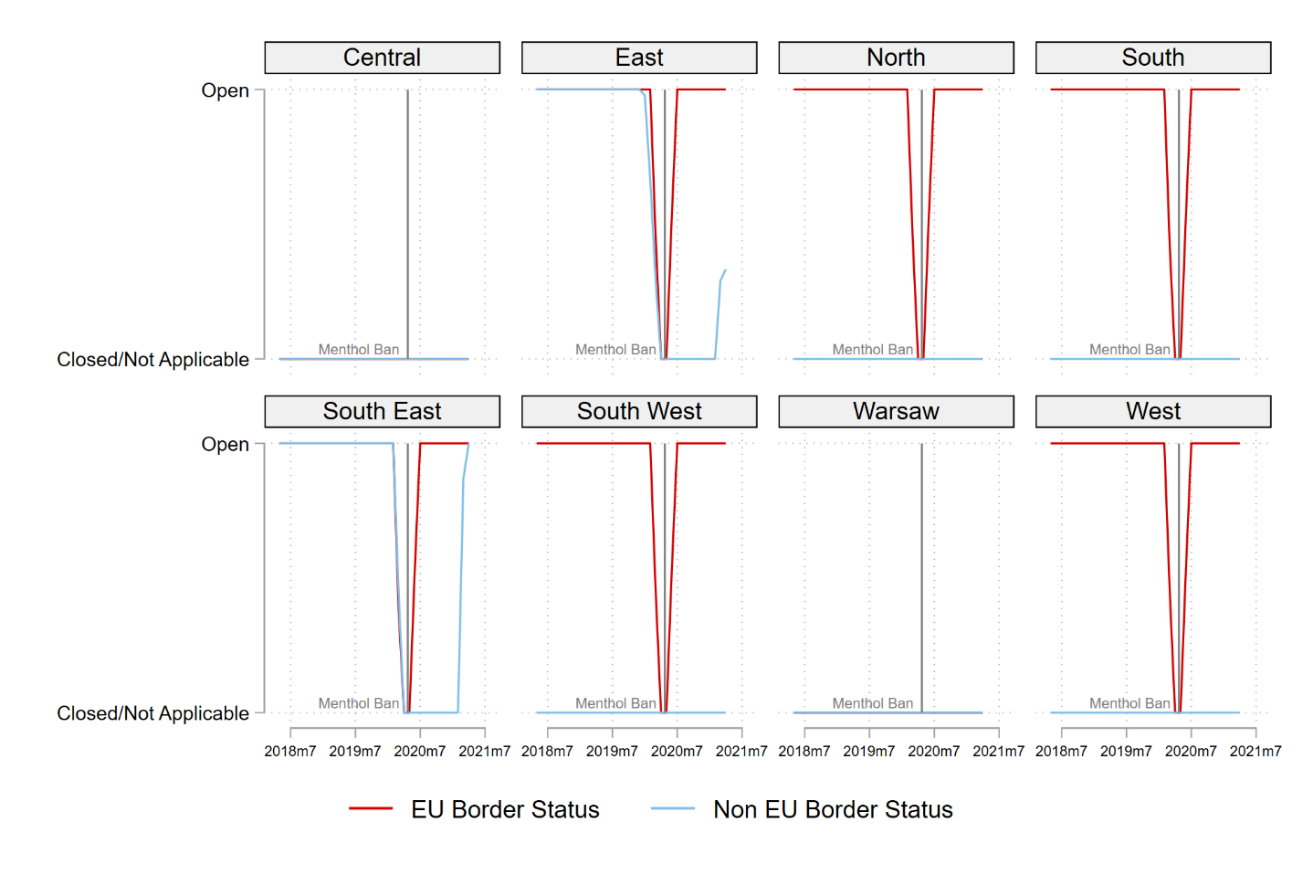


Note: Open has a numeric value of 1 and Closed/Not Applicable has a numeric value of 0.

Supplemental Figure 2: Regional average cigarette price (Real PLN per stick, May 2018—April 2021)


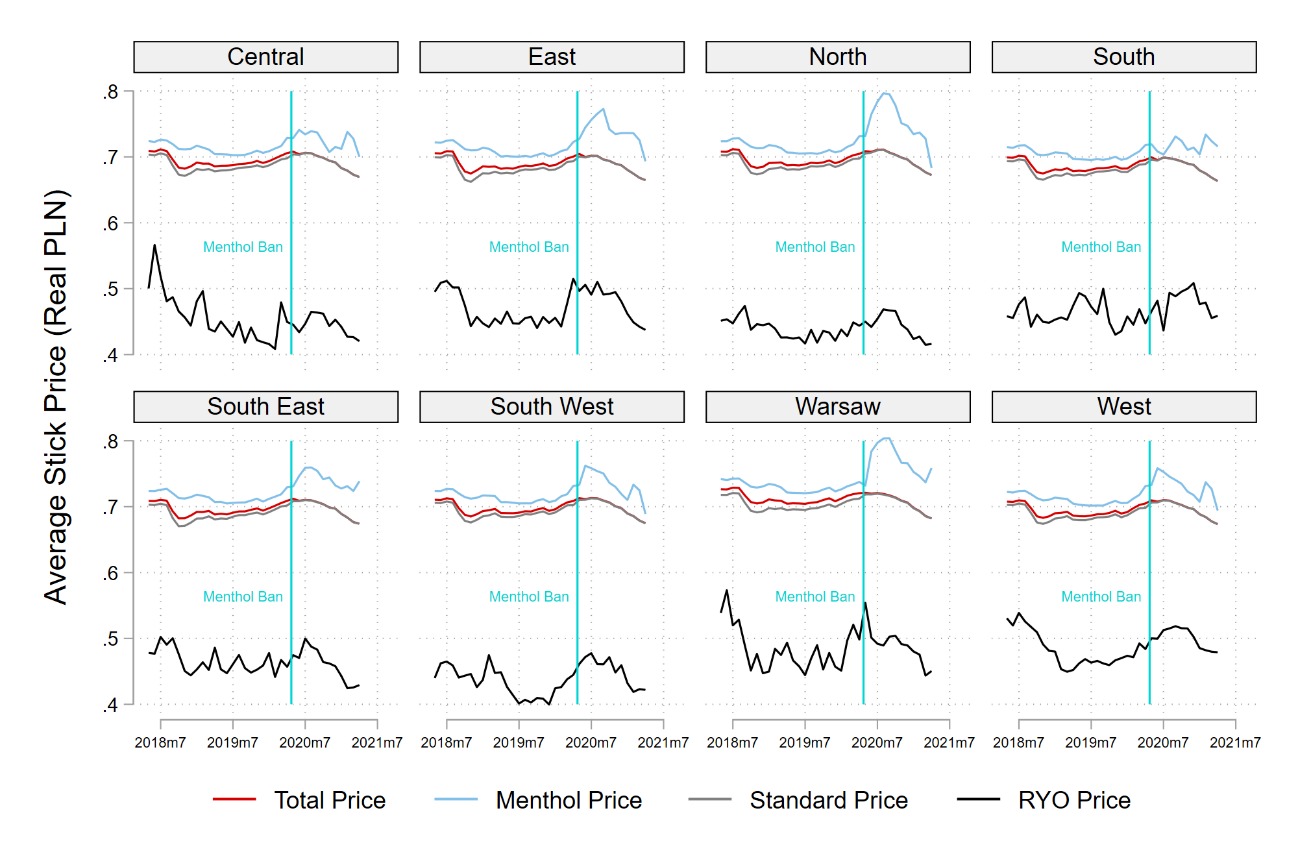


Note: Standard = Unflavored cigarettes

Supplemental Figure 3: Changes in Predicted Cigarette Sales Before and After the Menthol Ban Based on Pre-Ban Menthol Share


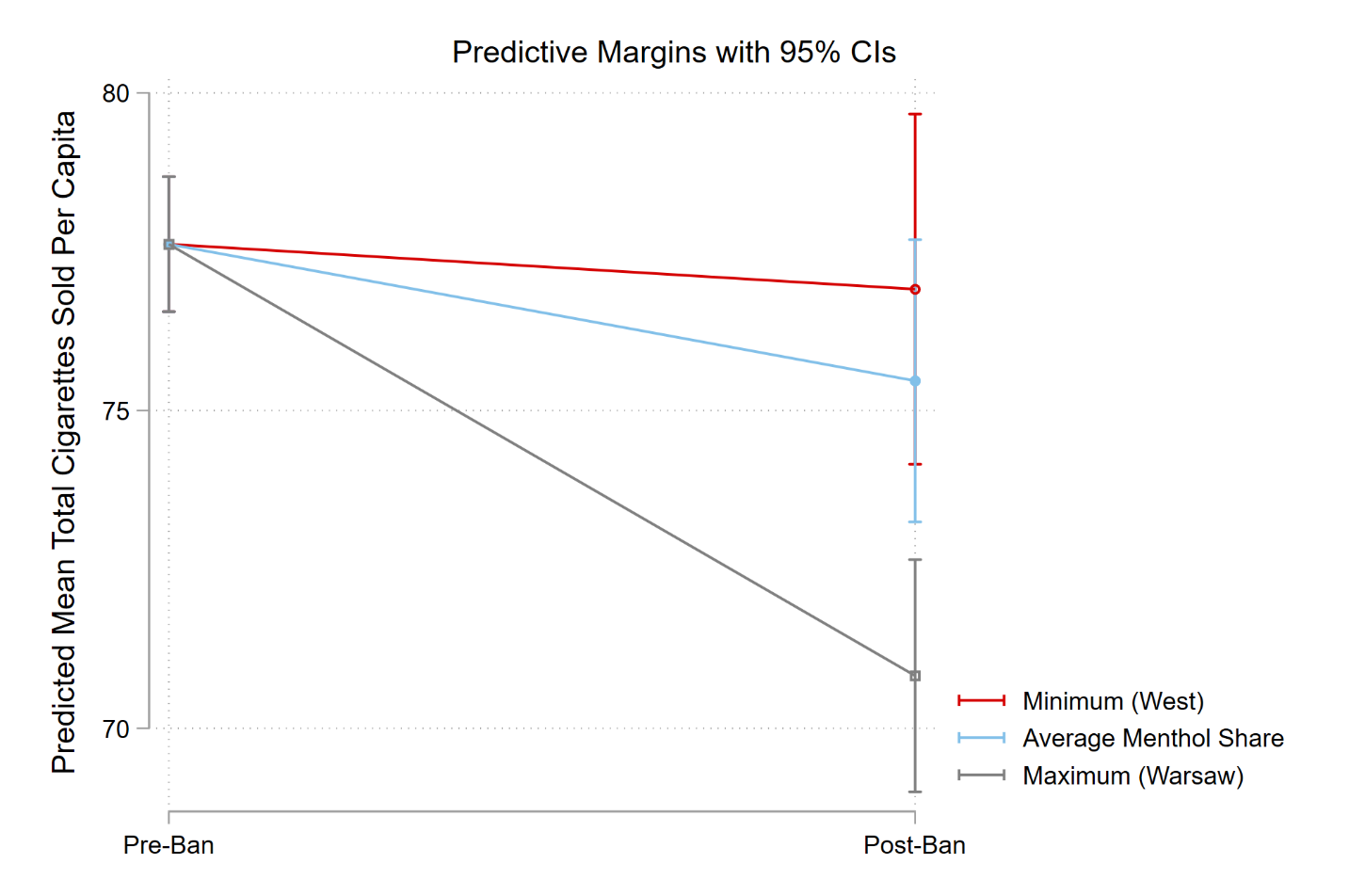


Supplemental Figure 4: Changes in Predicted RYO Tobacco Sales Before and After the Menthol Ban Based on Pre-Ban Menthol Cigarette Share


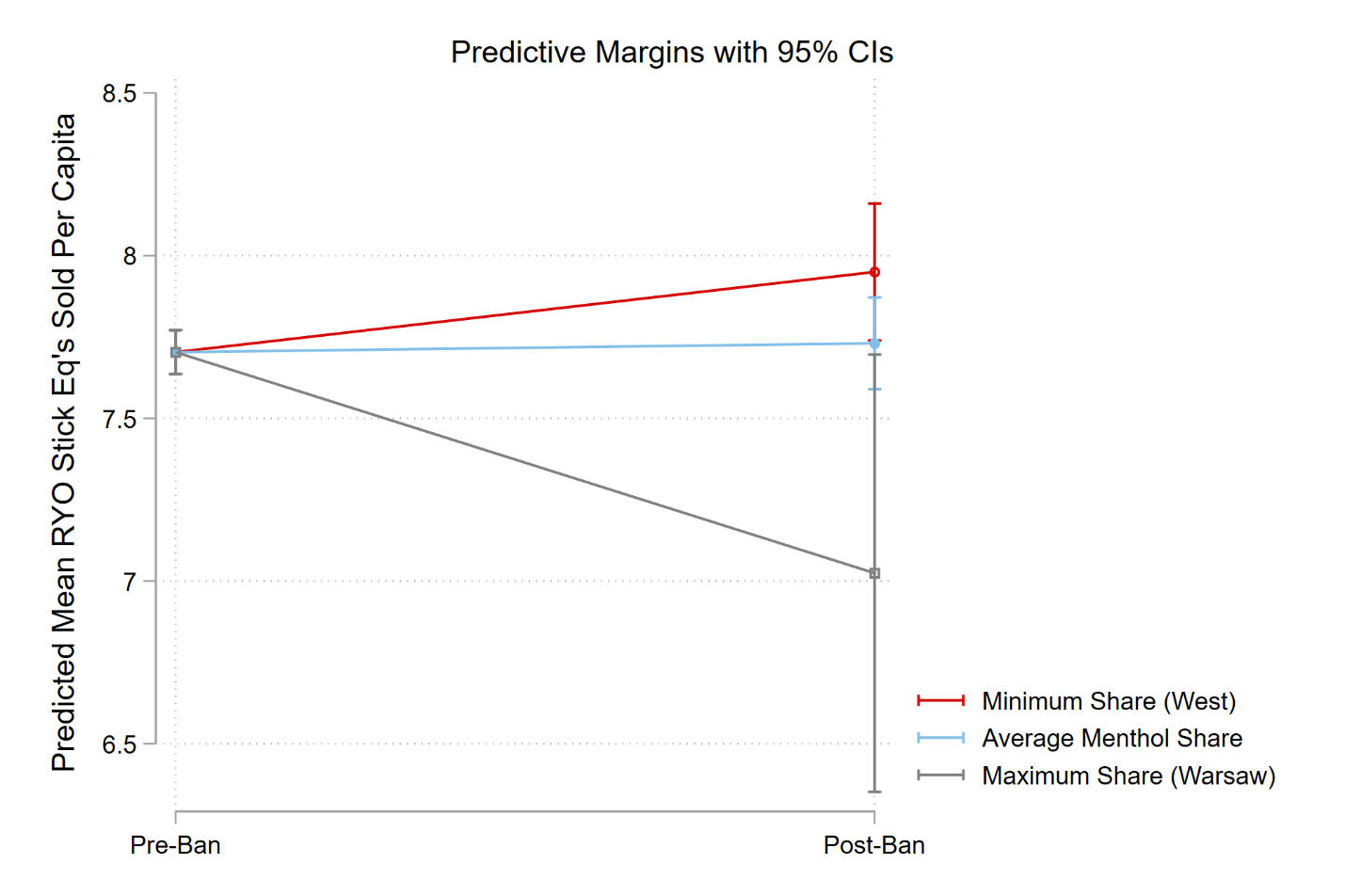


Supplemental Figure 5: Event Study for Cigarette Sales

Note: The dark blue line represents the interaction coefficient for each period in the event study while the light blue lines mark the edge of the 95% confidence interval for that coefficient. Estimated with all controls included in model 1 from Table 3.

Supplemental Figure 6: Event Study for RYO Tobacco Sales

Note: The dark blue line represents the interaction coefficient for each period in the event study while the light blue lines mark the edge of the 95% confidence interval for that coefficient. The model is estimated with all controls included in Model 3 from Table 3.

Supplemental Table 1: Cigarette Sales Model Building

|  | (1) | (2) | (3) | (4) | (5) | (6) | (7) |
| --- | --- | --- | --- | --- | --- | --- | --- |
|  | Base | +HDD | +EUBorder | +nonEUBorder | +Walking | +Employment | +Price |
| Post | 24.52^***^ | 21.30^**^ | 18.76^*^ | 17.23^***^ | 13.20^**^ | 12.87^*^ | 15.83^**^ |
| Pre-Menthol Share # Post | -19.80^**^ | -19.47^**^ | -18.87^**^ | -18.70^***^ | -15.59^***^ | -15.02^***^ | -16.86^***^ |
| Time Trend | -0.257^***^ | -0.0354 | 0.0664 | 0.0665 | 0.0840 | 0.0908 | 0.0314 |
| Heating Degree Days |  | -14.30^***^ | -15.18^***^ | -15.23^***^ | -13.98^***^ | -13.15^***^ | -14.26^***^ |
| EU Border Open |  |  | 9.213^***^ | 11.16^***^ | 9.284^***^ | 8.446^***^ | 4.528^**^ |
| Non-EU Border Open |  |  |  | -6.573 | -7.340^*^ | -6.766^*^ | -7.371^*^ |
| Walking Volume |  |  |  |  | 3.719^***^ | 3.695^***^ | 4.939^***^ |
| Employment Rate |  |  |  |  |  | 0.865^*^ | 1.380^***^ |
| Price |  |  |  |  |  |  | -7.594^***^ |
| Constant | 259.4^***^ | 105.2^*^ | 33.02 | 33.47 | 17.08 | -38.52 | 78.07 |
| Observations | 272 | 272 | 272 | 272 | 272 | 272 | 272 |
| AIC | 1759.9 | 1631.2 | 1603.9 | 1582.2 | 1573.6 | 1570.4 | 1550.0 |
| BIC | 1770.7 | 1645.6 | 1621.9 | 1603.8 | 1598.8 | 1595.7 | 1575.2 |
| Mean Menthol Ban Effect Size | 4.722 | 1.830 | -0.112 | -1.473 | -2.386 | -2.150 | -1.032 |
| Mean Menthol Ban Effect P-Value | 0.0003 | 0.149 | 0.949 | 0.407 | 0.164 | 0.199 | 0.561 |

Note: Region-fixed-effects coefficients are suppressed for space considerations. ^*^ *p* < 0.05, ^**^ *p* < 0.01, ^***^ *p* < 0.001

Supplemental Table 2: RYO Model Building

|  | (1) | (2) | (3) | (4) | (5) | (6) | (7) |
| --- | --- | --- | --- | --- | --- | --- | --- |
|  | Base | +HDD | +EUBorder | +nonEUBorder | +Walking | +Employment | +Price |
| Post | 2.999 | 2.830 | 2.860 | 2.596^*^ | 2.353^*^ | 2.312^*^ | 2.320* |
| Pre-Menthol Share # Post | -2.582 | -2.564 | -2.571 | -2.542^*^ | -2.354^*^ | -2.285^*^ | -2.288* |
| Time Trend | 0.0760^***^ | 0.0877^***^ | 0.0865^***^ | 0.0865^***^ | 0.0875^***^ | 0.0883^***^ | 0.0881*** |
| Heating Degree Days |  | -0.753^***^ | -0.742^***^ | -0.751^***^ | -0.675^***^ | -0.574^***^ | -0.575*** |
| EU Border Open |  |  | -0.109 | 0.226 | 0.113 | 0.0103 | 0.00536 |
| Non-EU Border Open |  |  |  | -1.133^*^ | -1.180^*^ | -1.110^*^ | -1.110* |
| Walking Volume |  |  |  |  | 0.225 | 0.222 | 0.222 |
| Employment Rate |  |  |  |  |  | 0.105 | 0.107 |
| Price |  |  |  |  |  |  | -0.118 |
| Constant | -50.42^***^ | -58.53^***^ | -57.68^***^ | -57.60^***^ | -58.59^***^ | -65.37^***^ | -65.23^***^ |
| Observations | 272 | 272 | 272 | 272 | 272 | 272 | 272 |
| AIC | 518.3 | 491.1 | 492.9 | 451.4 | 451.0 | 448.1 | 448.1 |
| BIC | 529.1 | 505.6 | 510.9 | 473.0 | 476.2 | 473.3 | 473.3 |
| Mean Menthol Ban Effect Size | 0.418 | 0.265 | 0.288 | 0.0536 | -0.00162 | 0.0272 | 0.0326 |
| Mean Menthol Ban Effect P-Value | 0.00639 | 0.101 | 0.0856 | 0.597 | 0.989 | 0.798 | 0.841 |

Note: Region-fixed-effects coefficients are suppressed for space considerations. ^*^ *p* < 0.05, ^**^ *p* < 0.01, ^***^ *p* < 0.001
